# Procalcitonin to reduce exposure to antibiotics and individualise treatment in hospitalised old patients with pneumonia: a randomised study

**DOI:** 10.1186/s12877-022-03658-4

**Published:** 2022-12-14

**Authors:** Gaëtan Gavazzi, Sabine Drevet, Matthieu Debray, Jean Luc Bosson, Fatah Tidadini, Marc Paccalin, Benoit de Wazieres, Thomas Celarier, Marc Bonnefoy, Virginie Vitrat

**Affiliations:** 1 CHU Grenoble Alpes, B - Hôpital Nord, Av. des Maquis du Grésivaudan Service Universitaire de Gériatrie Clinique, La Tronche, 38700 Grenoble, France; 2 T -Raig, TIMC-IMAG, UMR 5525 Université Grenoble Alpes, Grenoble, France; 3 Gérontopole AURA, Saint-Etienne, France; 4 Centre Hospitalier Annecy Genevois, Pringy Metz-Tessy, France; 5 MESP TIMC-IMAG UMR 5525, Université Grenoble Alpes/CNRS, Grenoble INP, Grenoble, France; 6 Pôle de Santé Publique, CHU Grenoble Alpes, Grenoble, France; 7 Département de chirurgie générale et digestive, CHU Grenoble Alpes, Grenoble, France; 8 Pôle de Gériatrie, CHU de Poitiers, Poitiers, France; 9 Service de médecine interne et gériatrique, CHU de Nîmes, Nîmes, France; 10 Chaire Santé des Ainés-Université Jean Monnet, Saint-Etienne, France; 11 Service de Gérontologie Clinique, CHU de Saint-Etienne, Saint-Etienne, France; 12 Service de médecine gériatrique, CHU Lyon, Groupement hospitalier Sud, Pierre-Bénite, France; 13 Inserm 1060-CarMeN, Oullins, France

**Keywords:** Aged, Antibiotic duration, Bacterial infection biomarkers, Pneumonia, Procalcitonin

## Abstract

**Background:**

Treating pneumonia in old patients remains challenging for clinicians. Moreover, bacterial antimicrobial resistance is a major public health threat.

**Objective:**

The PROPAGE study evaluated the interest of a strategy using serial measurements of procalcitonin (PCT) to reduce the duration of antibiotic therapy in old patients with pneumonia.

**Methods:**

PROPAGE took place from Dec.-2013 to Jun.-2016 in eight French geriatric units. It was a prospective, comparative, randomised, open-label study involving old patients (≥ 80 years) who had initiated antibiotic treatment for pneumonia in the previous 48 h. PCT was monitored in all patients and two decision-making PCT-based algorithms guided antibiotic therapy in patients from the PCT group.

**Results:**

107 patients were randomised (PCT, *n* = 50; Control, *n* = 57). Antibiotic therapy exposure was reduced in the PCT group as compared to the Control group (median duration of antibiotic therapy, 8 vs. 10 days [rank-test, *p* = 0.001]; antibiotic persistence rates on Days 6 and 8, 54% and 44% vs. 91% and 72%) and no significant difference was found in recovery rate (84% vs. 89.5%; Pearson Chi² test, *p* = 0.402).

**Conclusion:**

Although, the superiority of the strategy was not tested using a composite criterion combining antibiotic therapy duration and recovery rate was not tested due to the small sample size, the present study showed that monitoring associated with PCT-guided algorithm could help shorten antibiotic treatment duration in the very old patients without detrimental effects. Measuring PCT levels between Day 4 and Day 6 could be helpful when making the decision regarding antibiotic discontinuation.

**Trial registration:**

NCT02173613. This study was first registered on 25/06/2014.

**Supplementary Information:**

The online version contains supplementary material available at 10.1186/s12877-022-03658-4.

## Introduction

The rate of hospitalisations due to infectious diseases rises with age, reaching its highest incidence in those above 85 years of age. According to a recent systematic review, in industrialised countries, hospitalisations due to pneumonia have more than doubled in each consecutive age range going from 6.8, to 16.4, and to 34.6 episodes/1000 persons/year in patients aged 65–74, 75–84, and > 85 years, respectively [1]. A review focused on European countries shows similar increases in incidence with age despite the use of a 23-valent pneumococcal polysaccharide vaccine [2]. Additionally, the global burden of disease study from 2016 found that although pneumonia mortality rates have remained stable in most of the world, the numbers of pneumonia, hospitalisations, and deaths have increased as the amount of older adults has doubled between 1990 and 2016 [3].

Infectious diseases such as pneumonia are not only more frequent and severe in old people, but also have different characteristics [4]. Diagnosis is more complex because patients may present with atypical pneumonia signs and symptoms such as delirium, falls, and decompensation of chronic diseases. It is also more difficult to obtain satisfactory microbiological samples, chest X-rays, and computerised tomography (CT) scans to reach a definite diagnosis [4, 5]. Diagnostic uncertainty often prompts the prescription of empiric antibiotic treatments as physicians balance the risks and benefits of initiating or withholding antibiotics in older patients [4, 6]. Between 2000 and 2018, global antibiotic use increased by 46% [7]. According to a large study (*n* = 6481) in the United States, two-thirds of patients hospitalised with pneumonia received excess antibiotic therapy. Notably, each additional day of antibiotic therapy was associated with a 5% increase in the risk of antibiotic-associated adverse events [8]. These elements underlie bacterial antimicrobial resistance (AMR) which is a major public health threat. In 2019, lower respiratory infections alone resulted in over 1.5 million deaths associated with AMR, consequently becoming the most burdensome infectious disease [9].

In France, excess antibiotic use both in hospitals and in the community is also a remarkable problem. Mean antibiotic consumption rates are consistently above the European average despite national healthcare system campaigns to decrease antibiotic overuse. However, these campaigns were not as successful as expected in old people possibly because comorbidities which are frequent in this population trigger empiric antibiotic treatments [10]. Despite recommendations indicating a minimum of 5 days of antibiotic treatment for pneumonia and additional days depending on the patient’s clinical stability [11], it is not uncommon for physicians to prescribe a standard antibiotic treatment lasting 10 to 14 days [12]. Old people are particularly vulnerable to the deleterious effects of excessive antibiotic use partly due to age-related drug metabolism changes resulting in more severe and frequent adverse reactions, drug interactions, multi-drug resistant organisms, *Clostridium difficile*, and microbiome alterations [4, 6].

The limitation of antibiotic use in old people is, therefore, a major issue. One way to prevent problems related to extended antibiotic use without altering patient prognosis is discontinuing antibiotic treatment early [11]. Immune response biomarkers such as procalcitonin (PCT), C-reactive protein, interleukin-6, and presepsin help monitor antibiotic efficacy and individualise treatment duration by minimising or optimising antibiotic use [13]. Among these, PCT is one of the most studied for guiding antibiotic treatment initiation and discontinuation in pneumonia and other acute infectious syndromes [14–19]. PCT may have immune-modulatory properties and its production increases throughout the body during bacterial infections [13, 17]. However, there are few studies in old people specifically assessing the role of PCT-based algorithms to guide antibiotic treatment discontinuation; in this age group, studies have been mostly conducted to assess PCT’s utility to identify infections rather than to individualise treatment duration [20–22]. Also, very old people are often not included in studies because of their many comorbidities even though they should be included since they are at considerable risk of inappropriate antibiotic treatment [23–25]. Thus, data to determine if PCT monitoring helps to reduce antibiotic use in old patients, in particular very old patients, with pneumonia is lacking. The present study aims to evaluate the utility of serial measurements of PCT and decision-making algorithms to guide antibiotic treatment duration decisions in old people with pneumonia compared to conventional treatment strategies.

## Material and methods

PROPAGE (*PROcalcitonine chez les Patients AGEs*) was an interventional, randomised, comparative, open-label study involving old patients admitted for pneumonia to eight geriatric units (six French hospitals).

Geriatricians and/or infectious disease specialists agreeing to participate in the study included all volunteer patients admitted in their unit provided (1) they were ≥ 80 years of age, (2) they had initiated antibiotic treatment for pneumonia in the previous 48 h, and (3) their PCT levels were evaluated prior to treatment initiation on Day 0. Pneumonia was previously defined by the presence of at least 2 clinical signs of pneumonia and based on the results of X-Ray or scanner. Patients were not included if (1) they had an infection due to a virus, parasite, *Listeria* spp., *Legionella pneumophilia*, or *Mycobacterium tuberculosis*; (2) they had an associated endovascular or chronic infection, or a lung abscess upon admission; (3) they had severe immunosuppression; (4) they were under palliative care; (5) they were deceased 24 h post-admission; or (6) they were receiving antibiotics for a chronic infection.

At inclusion, patients were randomised into two groups (1:1): i.e., PCT and Control groups. Randomisation was centralised via electronic case report forms (eCRFs) once eligibility was verified. Stratification was by centre and balanced by random size blocks.

PCT was assessed on patients from both the PCT and Control groups on Days 2, 4, 6, and 8 post-admission, and then after discharge or on Day 15 with the miniVidas® B.R.A.H.M.S PCT device (BioMérieux, S.A., Marcy L’Etoile, France) and PCT kits. PCT results were immediately transmitted to the physicians. They guided antibiotic treatment for patients in the PCT group, only. Patients assigned to the PCT group received an antibiotic regimen that was terminated early according to clinical evaluation algorithms guided by PCT levels. Patients assigned to the Control group received a conventional antibiotic regimen that was terminated according to treating physician’s discretion. Once discharged, patients were followed via telephonic interviews at 6 weeks (Week 6). Interviews were performed by the investigators.

**Fig. 1:**
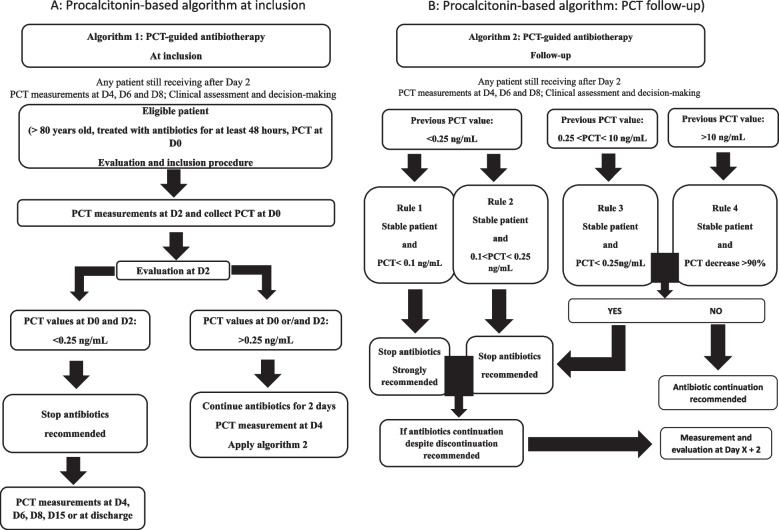
PCT-guided algorithms: (1) On Day 2 (2) After Day 2. PCT: procalcitonin

In the PCT group, single decision-making PCT-based approach with 2 consecutive algorithms to guide antibiotic therapy were used to assess patients (Fig. 1). Physicians were instructed on their use prior to study initiation. Algorithm 1 corresponded to the assessments and decisions on Day 2, and Algorithm 2 to the assessments and decisions on Days 4, 6, and 8. Treating physicians recommended antibiotic discontinuation depending on PCT levels on Days 2, 4, 6, and 8. In the Control group, patients were managed per usual treatment strategies according to the recommendations from the French Infectious Diseases Society (SPILF, *Société de pathologie infectieuse de langue française*) [26]; PCT measurements on Days 2, 4, 6 and 8 were also performed.

The PCT-based algorithms were considered useful if they significantly shortened antibiotic treatment duration. Treatment failure and recovery rates on Day 45 were determined in each group and compared. Infection relapse or death from any cause were considered therapeutic failures. Recovery rate was defined as complete if, according to the physician judgement, no clinical sign of pneumonia persisted; otherwise, recovery was partial.

Demographic data, nutritional (Mini Nutritional Assessment [MNA] [27]) and functional status (activities of daily living [ADLs] [28] and instrumental activities of daily living [IADLs] [29]), clinical data (including vital signs) and disease severity scores (Acute Physiology and Chronic Health Evaluation II [APACHE II] [30], pneumonia severity-of-illness index [PSI] [31], and confusion, uraemia, respiratory rate, blood pressure, age ≥ 65 years [CURB 65] [32]) were documented for all patients upon inclusion and retrospectively for functional status (ADL and IADL). Adverse events (AEs) were collected. Data were collected on the eCRFs by the investigators.

All statistical analyses were performed with Stata (StataCorp, Texas, USA). A descriptive statistical analysis at baseline was performed for all collected variables and results were reported as mean (standard deviation, SD) or median (interquartile ranges, IQR) if appropriate for continuous variables, and count (percentage, %) for qualitative variables. Usual parametric and non-parametric tests were used for group comparisons. All tests were two-sided at the 5% level of significance (*p* = 0.05). The main analysis was performed on the intention-to-treat (ITT) population. A secondary analysis was performed on the per protocol (PP) population. ITT population included all randomised patients complying with all inclusion criteria and none of the non-inclusion criteria with available PCT evaluation on Day 2; patients from the ITT population whose antibiotic treatment did not respect the PCT-based algorithms were excluded from the PP population.

PROPAGE was conducted in compliance with the International Conference on Harmonisation-Good Clinical Practice Guidelines and the applicable regulatory requirements. The protocol was authorised by the *Agence Nationale de Sécurité du Médicament* on 19 September 2011 and approved by the *Comité de Protection des Personnes Sud Est V* (Independent Ethics Committee) of the teaching hospital of Grenoble on 05 October 2011 (approval n°2011-A01026-35). All patients (or their legal representatives) gave their informed consent for participation before being included in the study (i.e., within the 48 h following the initiation of the antibiotic treatment). This trial is registered in ClinicalTrials.gov (NCT02173613, first posted date: 25/06/2014).

## Results

**Fig. 2:**
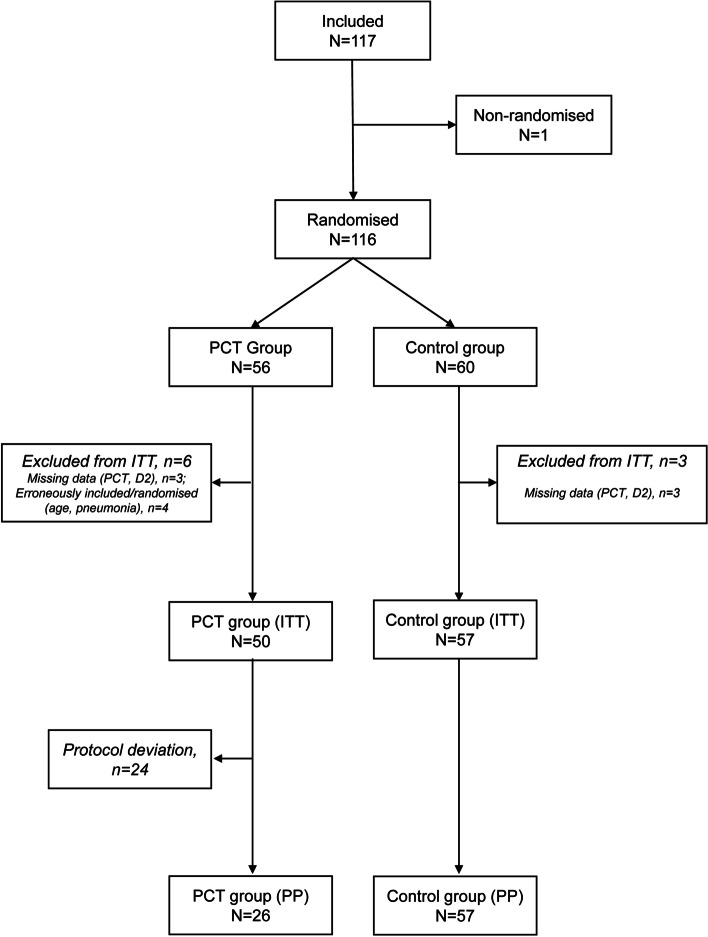
Study flow chart. D: day; ITT: intention-to-treat; n or N: number of subjects; PCT: procalcitonin; PP: per protocol

PROPAGE took place from December 2013 to June 2016. As shown in the participant flow diagram (Fig. 2), 117 patients were included and 116 randomised: 60 patients in the control group and 56 in the PCT group. Post-randomisation, three patients in the Control group and two patients in the PCT group were excluded from the randomised population as the PCT level was missing on Day 2. In addition, four patients in the PCT group were excluded as they were erroneously included in the study (e.g., non-confirmation of the diagnosis of pneumonia). Finally, 107 patients (50 in the PCT group and 57 in the Control group) were included in the ITT population. As 24 patients did not respect the PCT-based algorithms, the PP population included 83 patients: 26 and 57 for the PCT and Control groups, respectively. The compliance in the PCT group was of 52%.

As the study was randomized, no statistically significant difference was observed between groups in baseline characteristics (Table 1, ITT population). The patients were very old in both groups and evenly split in terms of gender. Most of them did not smoke. More than three-quarters of them resided in their own homes and the rest were nursing home residents. In both groups, most of them had a poor nutritional status or were at risk of malnutrition. The patient’s functional autonomy was in decline and their ADL scores generally worsened relative to 15 days prior to being hospitalised. In both groups, the vital signs, mental, and mortality risk characteristics of the patients were similar. Their blood pressure was slightly elevated (systolic 130 mmHg, diastolic 69 mmHg), their heart rate was in the upper limit of normal (80 vs. 83 bpm), their respiratory rate was slightly elevated (22.6 vs. 25 breaths/minute), 31% of the patients had an impaired mental status, the majority of patients were at intermediate (13%) or high risk (17%) of death per their CURB 65 scores, and their APACHE II scores indicated a mortality probability of 8‒15% as well. A quarter of the patients in either group had purulent sputum. The groups differed in terms of other elements specific to their pneumonia. Patients in the Control group had more expectoration, cough, bronchial congestion, and worse partial pressure of oxygen (PAO_2_), while the patients in the PCT group had higher leukocyte counts and slightly worse pain. Lastly, almost 80% and 84% of the patients in the Control and PCT groups had PSI scores indicating hospitalisation (IV and V), the rest had a score that indicated they could benefit from a brief hospitalisation (III). None of these differences was statistically significant. Finally, mean (SD) PCT levels measured before initiation of antibiotic therapy were 2.6 (6.70) ng/ml in the PCT and 4.8 (12.73) ng/ml in the Control group (Student t test, *p* = 0.25).

**Table 1:** Initial characteristics of patients included in each group of the randomised PROPAGE study (*N* = 107).

		Control group (*n* = 57)	PCT group (*n* = 50)	*p value*
Gender, N (%)	Female	29 (50.9%)	25 (50.0%)	*0.93*
	Male	28 (49.1%)	25 (50.0%)	
Age (years), mean (SD)		87.6 (5.0)	88.0 (5.1)	*0.66*
Residence, N (%)	Own home	44 (77.2%)	39 (79.6%)	*0.77*
	Nursing home	13 (22.8%)	10 (20.4%)	
Smoking status, N (%)	Yes	2 (3.6%)	2 (4.1%)	> 0.99*
	No	45 (81.8%)	40 (81.6%)	
	Former smoker	8 (14.5%)	7 (14.3%)	
MNA at inclusion, N (%)	Normal	1 (4.0%)	-	0.84*
	Risk of malnutrition	11 (44.0%)	8 (53.3%)	
	Poor nutritional status	13 (52.0%)	7 (46.7%)	
ADLs score on Day − 15, N (%)	0-1.5	3 (8.3%)	3 (8.1%)	> 0.99*
	2-3.5	8 (22.2%)	9 (24.3%)	
	4–6	25 (69.4%)	25 (67.6%)	
ADLs score on Day 0, N (%)	0-1.5	7 (20.6%)	6 (17.6%)	0.65*
	2-3.5	11 (35.3%)	8 (23.5%)	
	4–6	16 (47.1%)	20 (58.8%)	
IADL on Day − 15, N (%)	0–2	18 (50.0%)	13 (36.1%)	0.39*
	3–5	6 (16.7%)	5 (13.9%)	
	6–8	12 (33.3%)	18 (50.0%)	
Mental status, N (%)	Impaired	17 (31.5%)	15 (31.2%)	*0.98*
SBP (mmHg*)*, mean (SD)		130.7 (26.3)	130.8 (22.1)	*0.98*
DBP (mmHg*)*, mean (SD)		69.7 (14.3)	69.6 (13.0)	*0.96*
Heart rate (bpm*)*, mean (SD)		83.5 (18.1)	80.3 (17.2)	*0.35*
Respiratory rate (breath/min*)*, mean (SD)		22.6 (5.5)	25.0 (10.8)	*0.37*
PAO_2_ (mmHg*)*, mean (SD)		66.6 (23.4)	74.9 (31.4)	*0.31*
Leukocytes (/µl*)*, mean (SD)		9917 (5577)	10,971 (13,020)	*0.62*
Eosinophils (%)		1.4 (2.2)	1.8 (2.7)	*0.47*
PCT *(*ng/ml*)*, mean (SD)		4.84 (12.73)	2.6 (6.70)	*0.25*
Cough, N (%)		36 (65.4%)	26 (55.3%)	*0.30*
Bronchial congestion, N (%)		31 (57.4%)	21 (42.9%)	*0.14*
Chest pain, N (%)		2 (3.8%)	3 (6.1%)	0.67*
Purulent sputum, N (%)		13 (25.0%)	12 (25.0%)	*> 0.99*
History of pneumonia		48 (87.3%)	44 (88.0%)	> 0.99*
PSI (lowest risk class at least), N (%)	III	12 (21.0%)	8 (16.0%)	*0.07*
	IV	28(49.1%)	35 (70.0%)	
	V	17 (29.8%)	7 (14.0%)	
CURB 65 (lowest risk class at least), N (%)	1	8 (14.0%)	6 (12.0%)	0.83*
	2	24 (42.1%)	23 (46.0%)	
	3	25 (43.9%)	20 (40.0%)	
	4	-	1 (2.0%)	
APACHE II, mean (SD)		10.8 (3.9)	10.2 (3.6)	*0.47*

*ADL* Activities of daily living, *APACHE II* Acute Physiology and Chronic Health Evaluation II, *CURB 65* Confusion, uraemia, *DBP* Diastolic blood pressure, *IADL* Instrumental activities of daily living, *MNA* Mini Nutritional Assessment, *PCT* Procalcitonin, *PROPAGE* PROcalcitonine chez les Patients AGEs, PAO_2_Partial pressure of oxygen, *PSI* Pneumonia severity index, *RR* Respiratory rate, *SD* Standard deviation.

*Test of Fisher (otherwise Chi² or Student t test).

**Fig. 3:**
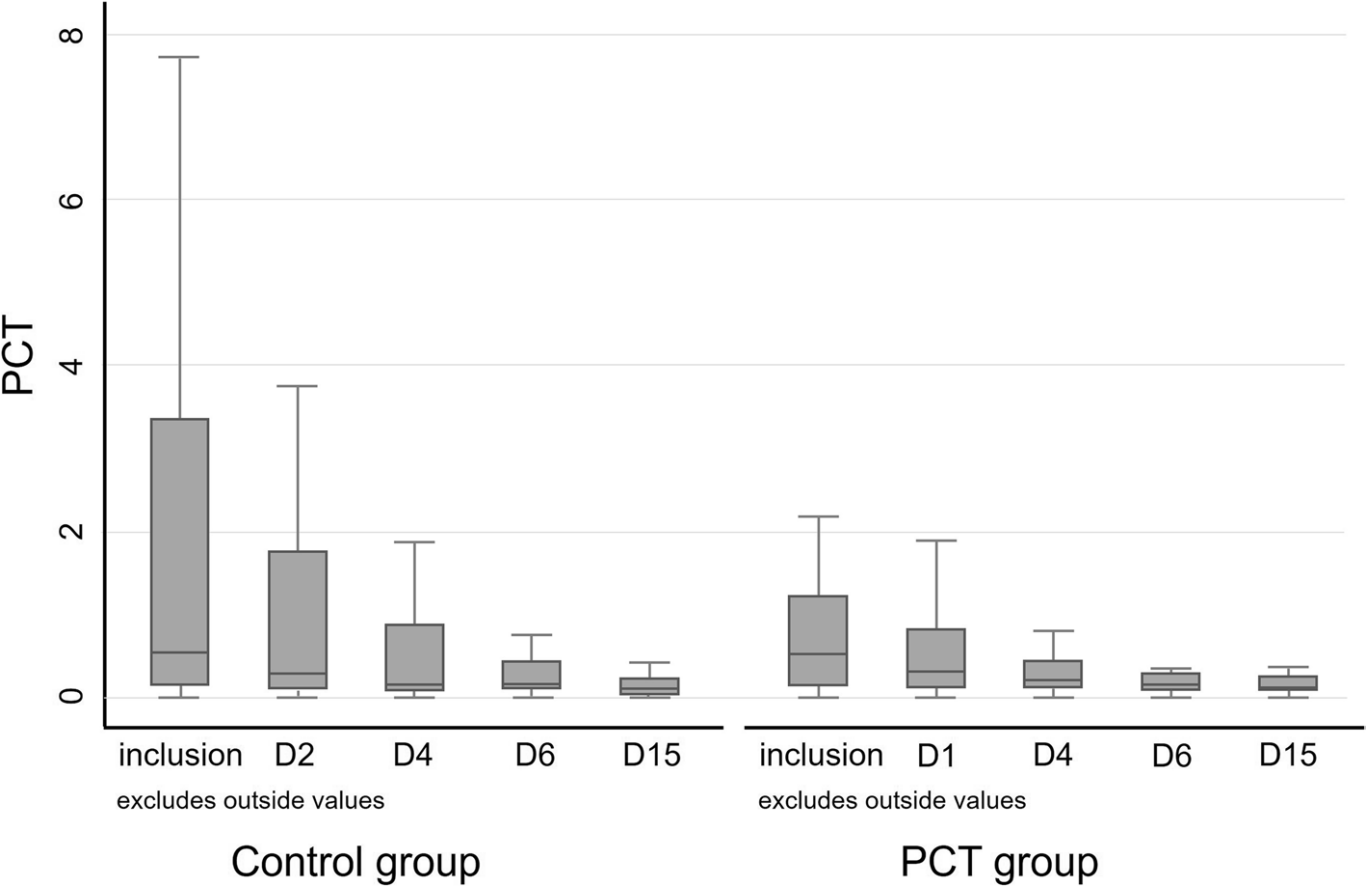
Box plot of PCT levels from inclusion to Day 15 by randomised group. J: day; PCT: procalcitonin

**Fig. 4:**
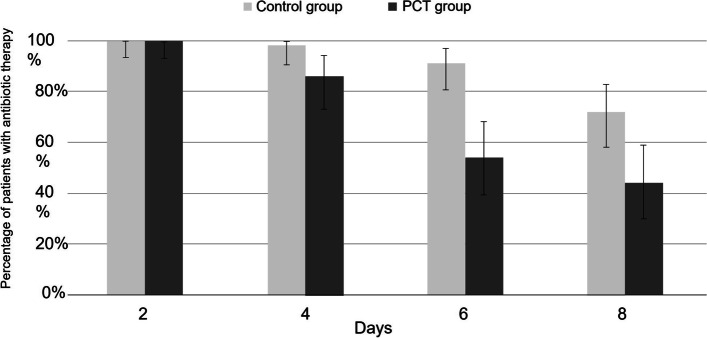
Percentage of patients exposed to antibiotic therapy per randomised group (*N*=107). D: day; PCT: procalcitonin

In the ITT population (main analysis), the median duration of antibiotic treatment in the PCT group was 8 days (IQR: 6–11 days), which was significantly shorter than in the Control group (10 days, IQR: 8–12; rank-test, *p* = 0.001). The recovery rate on Day 45 in the PCT group (84%) was lower than that of the Control group (89.5%), but no statistically significant difference was observed in recovery rate between the two groups (Pearson Chi² test, *p* = 0.402). As no relapse was reported, mortality rates were 16% and 10.5% in the PCT and Control groups, respectively. Serum PCT levels decreased rapidly between inclusion and Day 4 in both groups and then reached a plateau. PCT levels and their evolution over time were not significantly different between the two groups (ANOVA, respectively *p* = 0.366 and *p* = 0.515) (Fig. 3). Antibiotic persistence rates were clearly reduced on Days 6 and 8 in the PCT group as compared to the Control group (54% and 44% vs. 91% and 72%, respectively) (Fig. 4).

In the PP population (secondary analysis), median antibiotic treatment duration in the PCT group was one day shorter than in the ITT population. It was 3 days shorter in the PCT than in the Control group: 7 days (IQR: 6–10 days) vs. 10 days (IQR: 8–12). The difference between the PCT and Control groups was statistically significant (rank-test, *p* < 0.001). The recovery rate in the PCT group was slightly higher in the PP than in the ITT population (86.2% vs. 84%) but remained lower than in the Control group (86.2% vs. 89.5%). PCT changes over time were similar than that in the ITT population and no statistically significant difference was observed between the PCT and Control groups (ANOVA, *p* = 0.2648).

Overall, 35 patients (20 in the PCT group and 15 in the Control group) reported at least one adverse event leading to death (14 patients, 8 and 6 in the PCT and Control groups, respectively) and/or requiring hospitalisation or prolongation of existing hospitalisation or leading to permanent or significant disability/incapacity (23 patients, 13 and 10). Of the 47 reported adverse events, 9, 9, 8, and 7 were respiratory, thoracic, or mediastinal disorders (6 and 3 in the PCT and Control groups, respectively), cardiac disorders (4 and 5), psychiatric disorders (6 and 2), and infections or infestations (3 and 4).

Regarding missing information, data completion rates at baseline were between 87% and 100% for most clinical parameters and 37% and 68% for three parameters (respiratory rate, PAO_2_, and eosinophil counts). The completion rates of disease severity questionnaires were between 66% (APACHE II) and 100%. Lastly, the completion rates of functional autonomy questionnaires were between 37% and 68%.

## Discussion

This study showed, for the first time, that the use of a PCT-based algorithm significantly reduced the exposition to antibiotic therapy in a very old, and disabled patient population hospitalised for pneumonia without affecting their recovery as compared to usual treatment strategies (as per National Recommendations) [26]. The algorithm-guided decisions helped reduce the duration of antibiotic treatment when they were not followed completely (ITT population) and they reduced it more when they were followed perfectly (PP population). Regarding recovery rate, non-inferiority could not be evaluated due to a too small sample of the included population, but evolution was not different comparing both groups. Although the evolution of PCT levels was not significantly different between the groups, interestingly, the reduced antibiotic persistence rates started between the 4th and the 6th day when the PCT levels were lowest in the PCT group.

In terms of magnitude of the reduction in treatment duration, these results are consistent with previously reported data despite the particularity of this trial as it only recruited very old people with high Fine and CURB-65 scores. The effectiveness of PCT-based algorithms to guide antibiotic pneumonia treatment has been demonstrated in several randomised trials performed in different patient populations and clinical settings, ranging from primary care to emergency departments [17].

Analysing 26 trials including 6708 patients with respiratory infections across different clinical settings, a recent Cochrane review [17] found that the use of PCT-based clinical algorithms resulted in a 2.4-day reduction in antibiotic exposure, a reduction in antibiotic-related side effects (16.3% versus 22.1%), and a decrease in mortality rate at 30 days (8.6% in the PCT-group [286 deaths in 3336 participants] vs. 10.0%, in the control group [336 deaths in 3372 participants]. No obvious difference was found in mortality rates between groups in the present study; however, considering that it had limited statistical power and was not designed to establish this endpoint, this finding could not be confirmed. Moreover, the recent and large meta-analysis by Heilman et al. [33] showed that PCT-guided antibiotic treatment in old people was associated with significantly reduced antibiotic exposures and no increase in mortality as it has been observed in younger people. However, none of the included studies was designed specifically to old to very old populations with geriatric indicators such as nutritional or functional status.

Randomised clinical trials differ with respect to cut-off PCT levels for discontinuation, populations enrolled, and antibiotic treatment duration in the control groups. The randomised-controlled study by Schuetz et al. (ProHOSP) [34] is the largest study to date to examine PCT for respiratory infections. It involved 1359 adults with lower respiratory tract infection (LRTI) presenting to emergency departments. In this study, patients from the intervention group were treated with antibiotics according to a PCT guidance algorithm, and those in the control group according to international guidelines. Clinicians were encouraged to stop antibiotics in patients with PCT levels < 0.25 µg/l. The mean duration of antibiotic exposure was significantly shorter (5.7 vs. 8.7 days) and the LRTI antibiotic prescription rates were significantly lower (75.4 vs. 87.7%) in the intervention group than in the control group without affecting mortality. The algorithm was overruled by clinicians based on their clinical judgement in 9.2% of cases. The randomised-controlled study by Bouadna et al. (PRORATA) [16] evaluated the usefulness of a PCT-based algorithm to guide antibiotic therapy prescription in 621 unselected intensive care unit (ICU) patients with suspected bacterial infection. The cut-off level for discontinuation was < 0.5 µg/l or a decrease from maximal PCT recorded level by ≥ 80%; the PCT group had a 23% relative reduction in days of antibiotic exposure (11.6 vs. 14.3 days). The study showed non-inferiority for 28-day and 60-day mortality. The algorithm was overruled in 53% of cases.

The particularity of the present study lies in the population enrolled because it represents a typical population of short-stay patients hospitalised in France in Geriatric Units. They were very old (≥ 80 years), disabled, vulnerable, with poor nutritional status or at risk of malnutrition. As expected, most of the population exhibited impaired cognitive function, declined functional status, high severity scores, and respiratory decompensation upon their presentation. Notably, the atypical presentation of pneumonia and the difficulties in re-assessing the evolution of these patients often lead to inappropriate antibiotic initiation and longer antibiotic duration. Because of the presence of several comorbidities that blur infection status and hamper clinicians’ ability to make an accurate clinical assessment and correct diagnosis, prolongation of antibiotic treatment is frequent as was the case in the present study. Moreover, prolonged antibiotic treatment underlies adverse reactions and antibiotic resistance [35]. Antibiotic pressure is the key factor in promoting antibiotic resistance and the emergence of superinfection by multidrug-resistant bacteria which are particularly hazardous in old people [36]. In this context, PCT may guide antibiotic prescription to develop an individualised approach to antibiotic treatment duration according to the patients’ clinical responses [35]. In light of these data, some recommendations regarding frequency of PCT measurements could emerge and it could be considered helpful to measure PCT levels between Day 4 and Day 6 after inclusion when making the decision regarding antibiotic discontinuation.

There are some limitations to this study that clinicians should be aware of before using PCT in their own practice. The small population size and the short follow-up period (45 days) may have impacted the generalisation of the results due to possible uncontrolled bias resulting from the very particular study population. Although this patient population is representative of day-to-day care, larger and longer-term studies are needed to examine the way PCT is being used and its impact in real-world settings in this highly specific population. The small population size was responsible for the abandonment of the initial primary objective of the study. Indeed, the primary objective was initially to demonstrate that, as compared with the usual antibiotic treatment strategy, the strategy based on PCT monitoring shortened antibiotic treatment duration with at least equivalent recovery rates. The primary evaluation criterion was thus composite combining a superiority test for antibiotic therapy duration and a non-inferiority test for recovery rate. However, with an attrition rate of 10%, 340 patients were to be included in the present study to demonstrate a recovery rate of 84% in each group and a non-inferiority threshold of 10% for a 3-day difference in antibiotic therapy durations between the two groups with a power of 80% and an alpha-risk of 5%. Even after a 12-month extension of the recruitment period, only 117 patients could have been included in the present study. No hypothesis was therefore tested, and data were analysed as presented in “Material and Methods”. In addition, non-compliance rate was high (48%). At the time of the study, PCT assays were rarely performed, which probably partly explains the small sample size, and encourages us to emphasise on the need of assessing PCT levels to follow antibiotic treatment. Moreover, physicians were not used to guide antibiotic treatment on PCT levels and could be afraid to use the method including despite having agreed to participate in the study. This could be particularly true as the in-force recommendation stated to continue antibiotic therapy until recovery and as included patients were frail. This possibly explained both the small sample size and the high non-compliance rate. However, it was in accordance with that reported in previous trials [17]. This value is much higher than the one reported in the PROHOSP study (9.2%), a bit higher than the one described in the proREAL study (36.3%) [37] but equivalent to the one observed in the PRORATA study during which the algorithm was overruled in 53% of cases [16]. Physicians’ education and close guidance on how to apply the PCT-based algorithm seems necessary. Even though the population studied was not an ICU population, it exhibited several common characteristics: enrolment of very old patients with delirium, cardiac insufficiency or other organ decompensation further complicated the process of accurate clinical assessment to decide antibiotic discontinuation based on the sole clinical judgement.

The present algorithm could be used in viral pneumonia (i.e., pneumonia due to respiratory syncytial virus, metapneumovirus, coronavirus, or SARS-CoV-2) with suspicion of bacterial coinfections to promote early discontinuation of antibiotics. Recent studies have showed that in COVID-19 patients, antibiotic inappropriateness was very high, suggesting that PCT or PCT-changes could be used during the first 3 days from admission to help physician to decide (or not) antibiotic therapy initiation [38, 39].

## Conclusion

Finally, the present study showed that a PCT-guided algorithm could help reduce antibiotic treatment duration (from 10 to 8 days) in very old patients without detrimental effects. Identifying and diagnosing pneumonia in very old patients remain major challenges for clinicians as this patient population often presents atypically clinical signs. Facing these difficulties and considering the obvious risk of delaying antibiotic therapy in this high-risk population of patients, physicians usually prescribe antibiotic treatment. This PCT-guided algorithm could help physicians in their decision to stop antibiotic treatment, avoiding needlessly prolonged antibiotic treatment and therefore participating in the prevention of AMR. Further researches to determine the optimal PCT algorithm used in different situations and infectious syndromes are sorely needed in this very old patient population. Moreover, it seems necessary to disseminate information on the use of PCT-guided algorithm to effectively raise people’s awareness of this practice and improve their adherence.

## Supplementary Information

**Additional file 1.**

**Additional file 2.**

## Data Availability

The datasets used and/or analysed during the current study are available from the corresponding author on reasonable request.

## Abbreviations

ADL: activities of daily living
AE: adverse event
AMR: antimicrobial resistance
ANOVA: analysis of variance
APACHE II: Acute Physiology and Chronic Health Evaluation II
BP: blood pressure
CT: computerised tomography
CURB 65: confusion, uraemia, respiratory rate
DBP: diastolic blood pressure
Di: day i
eCRF: electronic case report form
IADL: instrumental activities of daily living
IQR: interquartile range
ITT: intention-to-treat
LRTI: lower respiratory tract infection
MNA: Mini Nutritional Assessment
PAO2: partial pressure of oxygen
PCT: procalcitonin
PROPAGE: PROcalcitonine chez les Patients AGEs
PSI: pneumonia severity index
SBP: systolic blood pressure
SD: standard deviation
SPILF: Société de Pathologie Infectieuse de Langue Française
