# Correction: Procalcitonin to reduce exposure to antibiotics and individualise treatment in hospitalised old patients with pneumonia: a randomised study

**DOI:** 10.1186/s12877-023-03818-0

**Published:** 2023-03-30

**Authors:** Gaëtan Gavazzi, Sabine Drevet, Matthieu Debray, Jean Luc Bosson, Fatah Tidadini, Marc Paccalin, Benoit de Wazieres, Thomas Celarier, Marc Bonnefoy, Virginie Vitrat

**Affiliations:** 1 CHU Grenoble Alpes, B - Hôpital Nord, Av. des Maquis du Grésivaudan Service Universitaire de Gériatrie Clinique, La Tronche, 38700 Grenoble, France; 2 T -Raig, TIMC-IMAG, UMR 5525 Université Grenoble Alpes, Grenoble, France; 3 Gérontopole AURA, Saint-Etienne, France; 4 Centre Hospitalier Annecy Genevois, Pringy Metz-Tessy, France; 5 MESP TIMC-IMAG UMR 5525, Université Grenoble Alpes/CNRS, Grenoble INP, Grenoble, France; 6 Pôle de Santé Publique, CHU Grenoble Alpes, Grenoble, France; 7 Département de chirurgie générale et digestive, CHU Grenoble Alpes, Grenoble, France; 8 Pôle de Gériatrie, CHU de Poitiers, Poitiers, France; 9 Service de Médecine Interne et Gériatrique, CHU de Nîmes, Nîmes, France; 10 Chaire Santé des Ainés-Université Jean Monnet, Saint-Etienne, France; 11 Service de Gérontologie Clinique, CHU de Saint-Etienne, Saint-Etienne, France; 12 Service de Médecine Gériatrique, CHU Lyon, Groupement Hospitalier Sud, Pierre-Bénite, France; 13 Inserm 1060-CarMeN, Oullins, France

**Correction: BMC Geriatrics 22, 965 (2022)**

**https://doi.org/10.1186/s12877-022-03658-4**

After publication of this article [1], the authors reported that in Fig. 4, the legend of the PCT and control groups have been inverted; the figure should have appeared as shown below.

**Fig. 4:**
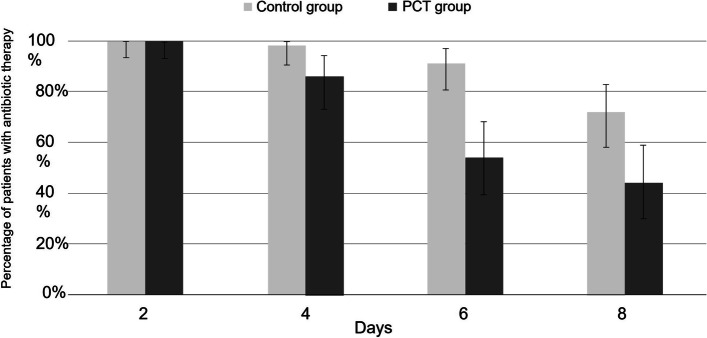
Percentage of patients exposed to antibiotic therapy per randomised group (*N*=107). D: day; PCT: procalcitonin

The original article [1] has been corrected.
