# Supplementary material for: Procalcitonin to reduce exposure to antibiotics and individualise treatment in hospitalised old patients with pneumonia: a randomised study
Source: BMC Geriatr. 2022 Dec 14;22:965. doi: 10.1186/s12877-022-03658-4 (PMC9748380; doi:10.1186/s12877-022-03658-4)
Supplement: Supplementary file 1 — Additional file 1. [file 12877_2022_3658_MOESM1_ESM.pdf]

We're building a better ClinicalTrials.gov. Check it out and tell us what you think!

U.S. National Library of Medicine

ClinicalTrials.gov

Find Studies
About Studies
Submit Studies
Resources
About Site
PRS Login

Reduction the Duration of Antibiotic Therapy in the Elderly (PROPAGE) (PROPAGE)

The safety and scientific validity of this study is the responsibility of the study sponsor and investigators. Listing a study does not mean it has been evaluated by the U.S. Federal Government. Read our disclaimer for details.

ClinicalTrials.gov Identifier: NCT02173613

Recruitment Status : Terminated
First Posted : June 25, 2014
Last Update Posted : May 2, 2022

Sponsor:
University Hospital, Grenoble

Information provided by (Responsible Party):

University Hospital, Grenoble

Study Details | Tabular View | No Results Posted | Disclaimer | How to Read a Study Record

Study Description

Go to

Brief Summary:

The main objective is to evaluate the interest of the repeated measurement of procalcitonin in patients with pulmonary infection to reduce the duration of antibiotic therapy in comparison with a conventional clinical strategy.

Condition or disease	Intervention/treatment	Phase
Lung Infection	Other: procalcitonine	Not Applicable

Study Design

Go to

Study Type :	Interventional (Clinical Trial)
Actual Enrollment :	117 participants
Allocation:	Randomized
Intervention Model:	Parallel Assignment
Masking:	None (Open Label)
Primary Purpose:	Other
Official Title:	Reduction of the Duration of Antibiotic Guided by Procalcitonin in Infections Lungs of Hospitalized Elderly: a Randomized
Actual Study Start Date :	August 2012
Actual Primary Completion Date :	December 2015
Actual Study Completion Date :	March 2016

Resource links provided by the National Library of Medicine

NIH NLM

MedlinePlus related topics: Antibiotics

U.S. FDA Resources

Arms and Interventions

Go to

Arm	Intervention/treatment
Experimental: Procalcitonine<br>every 2 days, they will receive the dose of PCT and decide to stop antibiotic treatment according to the algorithm 2. They will notify the results of clinical evaluations in the electronics and all adverse event report forms.	Other: procalcitonine<br>The recommendations will be based on the level of PCT: 4 levels of advice will be given:<br>• It is highly recommended to stop antibiotics if PCT <0.1ng/ml, and the recommended stop if 0.1ng/ml <PCT <0.25 ng / ml.<br>• It is recommended to continue treatment if 0.25 ng / ml <PCT ng / ml.<br>• Finally, if the initial PCT greater than 10 ng / ml, a stop will be advised in case of reduction to less than 10% of baseline level.
No Intervention: contrôle<br>Only clinical reassessments will be conducted and documented. Data on antibiotic will be listed and all adverse events. Data on the PCT from D2 to D4, D6, D8 and D15 output or will not be available to the prescriber.	

Outcome Measures

Go to

Primary Outcome Measures :

1. Duration of antibiotic therapy [Time Frame: Success of antibiotic therapy within 45 days of inclusion]

Eligibility Criteria

Go to

Information from the National Library of Medicine

Choosing to participate in a study is an important personal decision. Talk with your doctor and family members or friends about deciding to join a study. To learn more about this study, you or your doctor may contact the study research staff using the contacts provided below. For general information, Learn About Clinical Studies.

Ages Eligible for Study:	80 Years and older (Older Adult)
Sexes Eligible for Study:	All
Accepts Healthy Volunteers:	No

Criteria

Inclusion Criteria:

- Age ≥ 80 years
- Started antibiotics for a chest infection
- Procalcitonin performed J0 antibiotic treatment
- Person affiliated to the social security

Exclusion Criteria:

- Patients with a documented infection with germs after Listeria spp, Legionella pneumophilia, Mycobacterium tuberculosis
- Patients with a documented infection with a virus or parasite (eg hemorrhagic fever, malaria)
- Patients with endovascular infection associated (endocarditis, pacemaker. Intravascular catheter)
- Patients with lung abscess associated upon entry Patients with a chronic infection associated
- Patients with severe immunosuppression (HIV or transplant)
- Palliative patient
- Death within 24 hours of admission to nursing units.
- Presence of antibiotic treatment for chronic infection.
- Patient under guardianship, curatorship or any other administrative or judicial action or deprivation of the right or freedom
- Patients hospitalized without their consent

Contacts and Locations

Go to

Information from the National Library of Medicine

NIH NLM

To learn more about this study, you or your doctor may contact the study research staff using the contact information provided by the sponsor.

Please refer to this study by its ClinicalTrials.gov identifier (NCT number): ***NCT02173613***

Locations

France

University Hospital Grenoble

Grenoble, Rhone Alpes, France, 38000

Sponsors and Collaborators

University Hospital, Grenoble

Investigators

Principal Investigator: Gaetan Gavazzi University Clinic of Geriatrics Medicine, Division of Medicine multidisciplinary CHU de Grenoble,

More Information

Go to

Publications:

Polton D, Sermet C. Le vieillissement de la population va-t-il submerger le système de santé ? Bulletin Epidémiologique Hebdomadaire. 2006; 5-6:49-52.

Gaymu J. Aspects démographiques du vieillissement; Bulletin Epidémiologique Hebdomadaire. 2006; 5-6:38-9.

Fein AM. Pneumonia in the elderly: overview of diagnostic and therapeutic approaches. Clin Infect Dis. 1999 Apr;28(4):726-9. Review.

van der Steen JT, Ooms ME, van der Wal G, Ribbe MW. Withholding or starting antibiotic treatment in patients with dementia and pneumonia: prediction of mortality with physicians' judgment of illness severity and with specific prognostic models. Med Decis Making. 2005 Mar-Apr;25(2):210-21.

High KP. Infection as a cause of age-related morbidity and mortality. Ageing Res Rev. 2004 Jan;3(1):1-14. Review.

El Solh AA, Aquilina AT, Gunen H, Ramadan F. Radiographic resolution of community-acquired bacterial pneumonia in the elderly. J Am Geriatr Soc. 2004 Feb;52(2):224-9.

Torres OH, Muñoz J, Ruiz D, Ris J, Gich I, Coma E, Gurguí M, Vázquez G. Outcome predictors of pneumonia in elderly patients: importance of functional assessment. J Am Geriatr Soc. 2004 Oct;52(10):1603-9.

Büla CJ, Ghilardi G, Wietlisbach V, Petignat C, Francioli P. Infections and functional impairment in nursing home residents: a reciprocal relationship. J Am Geriatr Soc. 2004 May;52(5):700-6.

Harbarth S. Nosocomial transmission of antibiotic-resistant microorganisms. Curr Opin Infect Dis. 2001 Aug;14(4):437-42. Review.

Fischer JE, Harbarth S, Agthe AG, Benn A, Ringer SA, Goldmann DA, Fanconi S. Quantifying uncertainty: physicians' estimates of infection in critically ill neonates and children. Clin Infect Dis. 2004 May 15;38(10):1383-90. Epub 2004 Apr 29.

Christ-Crain M, Stolz D, Bingisser R, Müller C, Miedinger D, Huber PR, Zimmerli W, Harbarth S, Tamm M, Müller B. Procalcitonin guidance of antibiotic therapy in community-acquired pneumonia: a randomized trial. Am J Respir Crit Care Med. 2006 Jul 1;174(1):84-93. Epub 2006 Apr 7.

Yoshikawa TT. Antimicrobial resistance and aging: beginning of the end of the antibiotic era? J Am Geriatr Soc. 2002 Jul;50(7 Suppl):S226-9. Review.

Bonomo RA. Multiple antibiotic-resistant bacteria in long-term-care facilities: An emerging problem in the practice of infectious diseases. Clin Infect Dis. 2000 Dec;31(6):1414-22. Epub 2000 Nov 29. Review.

Rooney PJ, O'Leary MC, Loughrey AC, McCalmont M, Smyth B, Donaghy P, Badri M, Woodford N, Karisik E, Livermore DM. Nursing homes as a reservoir of extended-spectrum beta-lactamase (ESBL)-producing ciprofloxacin-resistant Escherichia coli. J Antimicrob Chemother. 2009 Sep;64(3):635-41. doi: 10.1093/jac/dkp220. Epub 2009 Jun 23.

Chastre J, Wolff M, Fagon JY, Chevret S, Thomas F, Wermert D, Clementi E, Gonzalez J, Jusserand D, Asfar P, Perrin D, Fieux F, Aubas S; PneumA Trial Group. Comparison of 8 vs 15 days of antibiotic therapy for ventilator-associated pneumonia in adults: a randomized trial. JAMA. 2003 Nov 19;290(19):2588-98.

Agarwal G, Awasthi S, Kabra SK, Kaul A, Singhi S, Walter SD; ISCAP Study Group. Three day versus five day treatment with amoxicillin for non-severe pneumonia in young children: a multicentre randomised controlled trial. BMJ. 2004 Apr 3;328(7443):791. Epub 2004 Mar 16. Erratum in: BMJ. 2004 May 1;328(7447):1066.

Singh N, Rogers P, Atwood CW, Wagener MM, Yu VL. Short-course empiric antibiotic therapy for patients with pulmonary infiltrates in the intensive care unit. A proposed solution for indiscriminate antibiotic prescription. Am J Respir Crit Care Med. 2000 Aug;162(2 Pt 1):505-11.

Micek ST, Ward S, Fraser VJ, Kollef MH. A randomized controlled trial of an antibiotic discontinuation policy for clinically suspected ventilator-associated pneumonia. Chest. 2004 May;125(5):1791-9.

el Moussaoui R, de Borgie CA, van den Broek P, Hustinx WN, Bresser P, van den Berk GE, Poley JW, van den Berg B, Krouwels FH, Bonten MJ, Weenink C, Bossuyt PM, Speelman P, Opmeer BC, Prins JM. Effectiveness of discontinuing antibiotic treatment after three days versus eight days in

mild to moderate-severe community acquired pneumonia: randomised, double blind study. BMJ. 2006 Jun 10;332(7554):1355.

Gervaix A, Pugin J. [Usefulness of procalcitonin in adults and children]. Rev Med Suisse. 2005 Mar 30;1(13):872-4, 877. Review. French.

Galetto-Lacour A, Zamora SA, Gervaix A. Bedside procalcitonin and C-reactive protein tests in children with fever without localizing signs of infection seen in a referral center. Pediatrics. 2003 Nov;112(5):1054-60.

Christ-Crain M, Jaccard-Stolz D, Bingisser R, Gencay MM, Huber PR, Tamm M, Müller B. Effect of procalcitonin-guided treatment on antibiotic use and outcome in lower respiratory tract infections: cluster-randomised, single-blinded intervention trial. Lancet. 2004 Feb 21;363(9409):600-7.

Gervaix A, Galetto-Lacour A, Gueron T, Vadas L, Zamora S, Suter S, Girardin E. Usefulness of procalcitonin and C-reactive protein rapid tests for the management of children with urinary tract infection. Pediatr Infect Dis J. 2001 May;20(5):507-11.

Harbarth S, Holeckova K, Froidevaux C, Pittet D, Ricou B, Grau GE, Vadas L, Pugin J; Geneva Sepsis Network. Diagnostic value of procalcitonin, interleukin-6, and interleukin-8 in critically ill patients admitted with suspected sepsis. Am J Respir Crit Care Med. 2001 Aug 1;164(3):396-402.

Gendrel D, Raymond J, Coste J, Moulin F, Lorrot M, Guérin S, Ravilly S, Lefèvre H, Royer C, Lacombe C, Palmer P, Bohuon C. Comparison of procalcitonin with C-reactive protein, interleukin 6 and interferon-alpha for differentiation of bacterial vs. viral infections. Pediatr Infect Dis J. 1999 Oct;18(10):875-81.

Chirouze C, Schuhmacher H, Rabaud C, Gil H, Khayat N, Estavoyer JM, May T, Hoen B. Low serum procalcitonin level accurately predicts the absence of bacteremia in adult patients with acute fever. Clin Infect Dis. 2002 Jul 15;35(2):156-61. Epub 2002 Jun 17.

Simon L, Gauvin F, Amre DK, Saint-Louis P, Lacroix J. Serum procalcitonin and C-reactive protein levels as markers of bacterial infection: a systematic review and meta-analysis. Clin Infect Dis. 2004 Jul 15;39(2):206-17. Epub 2004 Jul 2. Review. Erratum in: Clin Infect Dis. 2005 May 1;40(9):1386-8.

Christ-Crain M, Müller B. Biomarkers in respiratory tract infections: diagnostic guides to antibiotic prescription, prognostic markers and mediators. Eur Respir J. 2007 Sep;30(3):556-73. Review.

Stolz D, Christ-Crain M, Bingisser R, Leuppi J, Miedinger D, Müller C, Huber P, Müller B, Tamm M. Antibiotic treatment of exacerbations of COPD: a randomized, controlled trial comparing procalcitonin-guidance with standard therapy. Chest. 2007 Jan;131(1):9-19.

Schuetz P, Christ-Crain M, Wolbers M, Schild U, Thomann R, Falconnier C, Widmer I, Neidert S, Blum CA, Schönenberger R, Henzen C, Bregenzer T, Hoess C, Krause M, Bucher HC, Zimmerli W, Müller B; ProHOSP study group. Procalcitonin guided antibiotic therapy and hospitalization in patients with lower respiratory tract infections: a prospective, multicenter, randomized controlled trial. BMC Health Serv Res. 2007 Jul 5;7:102.

Briel M, Schuetz P, Mueller B, Young J, Schild U, Nusbaumer C, Périat P, Bucher HC, Christ-Crain M. Procalcitonin-guided antibiotic use vs a standard approach for acute respiratory tract infections in primary care. Arch Intern Med. 2008 Oct 13;168(18):2000-7; discussion 2007-8. doi: 10.1001/archinte.168.18.2000.

Kristoffersen KB, Søgaard OS, Wejse C, Black FT, Greve T, Tarp B, Storgaard M, Sodemann M. Antibiotic treatment interruption of suspected lower respiratory tract infections based on a single procalcitonin measurement at hospital admission--a randomized trial. Clin Microbiol Infect. 2009 May;15(5):481-7. doi: 10.1111/j.1469-0691.2009.02709.x. Epub 2009 Mar 5.

Nobre V, Harbarth S, Graf JD, Rohner P, Pugin J. Use of procalcitonin to shorten antibiotic treatment duration in septic patients: a randomized trial. Am J Respir Crit Care Med. 2008 Mar 1;177(5):498-505. Epub 2007 Dec 20.

Hochreiter M, Köhler T, Schweiger AM, Keck FS, Bein B, von Spiegel T, Schroeder S. Procalcitonin to guide duration of antibiotic therapy in intensive care patients: a randomized prospective controlled trial. Crit Care. 2009;13(3):R83. doi: 10.1186/cc7903. Epub 2009 Jun 3.

Tang H, Huang T, Jing J, Shen H, Cui W. Effect of procalcitonin-guided treatment in patients with infections: a systematic review and meta-analysis. Infection. 2009 Dec;37(6):497-507. doi: 10.1007/s15010-009-9034-2. Epub 2009 Oct 13. Review.

Caterino JM, Scheatzle MD, Forbes ML, D'Antonio JA. Bacteremic elder emergency department patients: procalcitonin and white count. Acad Emerg Med. 2004 Apr;11(4):393-6.

Stucker F, Herrmann F, Graf JD, Michel JP, Krause KH, Gavazzi G. Procalcitonin and infection in elderly patients. J Am Geriatr Soc. 2005 Aug;53(8):1392-5.

Dwolatzky T, Olshtain-Pops K, Yinnon AM, Raveh D, Rogowski O, Shapira I, Rotstein R, Berliner S, Rudensky B. Procalcitonin in the elderly: normal plasma concentrations and response to bacterial infections. Eur J Clin Microbiol Infect Dis. 2005 Nov;24(11):763-5.

Bignardi GE, Dhar R, Heycock R, Bansal S, Majmudar N. Can procalcitonin testing reduce antibiotic prescribing for respiratory infections? Age Ageing. 2006 Nov;35(6):625-6. Epub 2006 Jul 5.

Charlson ME, Pompei P, Ales KL, MacKenzie CR. A new method of classifying prognostic comorbidity in longitudinal studies: development and validation. J Chronic Dis. 1987;40(5):373-83.

Guigoz Y, Vellas B, Garry PJ. Assessing the nutritional status of the elderly: The Mini Nutritional Assessment as part of the geriatric evaluation. Nutr Rev. 1996 Jan;54(1 Pt 2):S59-65. Review.

KATZ S, FORD AB, MOSKOWITZ RW, JACKSON BA, JAFFE MW. STUDIES OF ILLNESS IN THE AGED. THE INDEX OF ADL: A STANDARDIZED MEASURE OF BIOLOGICAL AND PSYCHOSOCIAL FUNCTION. JAMA. 1963 Sep 21;185:914-9.

Lawton MP, Brody EM. Assessment of older people: self-maintaining and instrumental activities of daily living. Gerontologist. 1969 Autumn;9(3):179-86.

Knaus WA, Draper EA, Wagner DP, Zimmerman JE. APACHE II: a severity of disease classification system. Crit Care Med. 1985 Oct;13(10):818-29.

Fine MJ, Auble TE, Yealy DM, Hanusa BH, Weissfeld LA, Singer DE, Coley CM, Marrie TJ, Kapoor WN. A prediction rule to identify low-risk patients with community-acquired pneumonia. N Engl J Med. 1997 Jan 23;336(4):243-50.

Responsible Party:	University Hospital, Grenoble
ClinicalTrials.gov Identifier:	NCT02173613 History of Changes
Other Study ID Numbers:	1120
First Posted:	June 25, 2014 Key Record Dates
Last Update Posted:	May 2, 2022
Last Verified:	February 2016

Keywords provided by University Hospital, Grenoble:

infection lung elderly patients

Additional relevant MeSH terms:

Infections
